# Virtual Patient-Specific Quality Assurance of IMRT Using UNet++: Classification, Gamma Passing Rates Prediction, and Dose Difference Prediction

**DOI:** 10.3389/fonc.2021.700343

**Published:** 2021-07-20

**Authors:** Ying Huang, Yifei Pi, Kui Ma, Xiaojuan Miao, Sichao Fu, Hua Chen, Hao Wang, Hengle Gu, Yan Shao, Yanhua Duan, Aihui Feng, Jiyong Wang, Ruxin Cai, Weihai Zhuo, Zhiyong Xu

**Affiliations:** ^1^ Shanghai Chest Hospital, Shanghai Jiaotong University, Shanghai, China; ^2^ Department of Radiotherapy, The First Affiliated Hospital of Zhengzhou University, Zhengzhou, China; ^3^ Varian Medical Systems (China), Beijing, China; ^4^ Department of Hematology, Western Theater General Hospital, Chengdu, China; ^5^ PingAn Health Technology Co. Ltd, Shanghai, China; ^6^ Key Laboratory of Nuclear Physics and Ion Beam Application Ministry of Education, Fudan University, Shanghai, China

**Keywords:** deep learning, radiotherapy, quality assurance, prediction model, dose difference

## Abstract

The dose verification in radiotherapy quality assurance (QA) is time-consuming and places a heavy workload on medical physicists. To provide a clinical tool to perform patient specific QA accurately, the UNet++ is investigated to classify failed or pass fields (the GPR lower than 85% is considered “failed” while the GPR higher than 85% is considered “pass”), predict gamma passing rates (GPR) for different gamma criteria, and predict dose difference from virtual patient-specific quality assurance in radiotherapy. UNet++ was trained and validated with 473 fields and tested with 95 fields. All plans used Portal Dosimetry for dose verification pre-treatment. Planar dose distribution of each field was used as the input for UNet++, with QA classification results, gamma passing rates of different gamma criteria, and dose difference were used as the output. In the test set, the accuracy of the classification model was 95.79%. The mean absolute error (MAE) were 0.82, 0.88, 2.11, 2.52, and the root mean squared error (RMSE) were 1.38, 1.57, 3.33, 3.72 for 3%/3mm, 3%/2 mm, 2%/3 mm, 2%/2 mm, respectively. The trend and position of the predicted dose difference were consistent with the measured dose difference. In conclusion, the Virtual QA based on UNet++ can be used to classify the field passed or not, predict gamma pass rate for different gamma criteria, and predict dose difference. The results show that UNet++ based Virtual QA is promising in quality assurance for radiotherapy.

## Introduction

Intensity-modulated radiation therapy (IMRT), a widely used treatment modality for cancer patients, provides highly conformal dose distribution to the target while sparing surrounding healthy tissues (1). Quality assurance is performed to confirm the accuracy of dose calculation, data transmission, linear accelerator performance, radiotherapy positioning, and dosimeter response accuracy (2–6). It is essential to ensure the reliability of treatment delivery and improve patient safety. The process commonly involves comparing the calculated dose distribution or fluence with the measured dose distribution or fluence (7). However, the implementation of patient-specific QA measurement is time-consuming and places a heavy workload on medical physicists (8). Additionally, the measurement work takes a lot of clinical treatment time, which is unrealistic for busy centers. Furthermore, the QA process needs to be accomplished prior to treatment. For those QA results which do not meet the predefined “pass” criteria, re-planning and verification will cause an inevitable treatment delay. Therefore, a more streamlined, less-resourced, and automated patient-specific QA method for dose verification is necessary for radiotherapy centers.

With the development of machine learning and deep learning and its application in QA results prediction, the efficiency of patient-specific QA is expected to be improved (9–16). Valdes, Lam, Li (9–12) etc. to established prediction models for the gamma passing rate (GPR) based on the complexity parameters of the TPS plan. Other researchers (13, 14) investigated the deep learning algorithms to establish GPR prediction models based on planar dose distribution. Granville (15) used support vector machines (SVMs) to classify cold, hot, and normal plans based on the plan complexity parameters and accelerator performance parameters. Li (12) discussed the prediction model of whether the plan could pass the threshold using machine learning method. The above researchers have developed an accurate prediction model of QA results, confirming the feasibility of using machine learning or deep learning for patient-specific QA.

Previous prediction models based on machine learning or deep learning were only the results of dose verification but could not provide detailed information of dose difference (9–18). Predicting the trend and position of dose difference is an important work in automatic patient-specific QA in the near future.

In this study, we proposed a novel QA prediction model based on UNet + + using the planar dose distribution as input. A model can (a) provide the classification results whether the field QA passes; (b) predict the GPRs of different gamma criteria; (c) predict the trend and position of dose difference. The prediction model allows physicists to pre-mark potentially failed fields in a proactive way, analyze dose difference simultaneously and reduce patient delays associated with unqualified measurements. Additionally, it could reduce patient-specific QA measurements to verify data transmission and delivery accuracy combination with other tools. The model is expected to be a practical clinical tool to perform patient-specific QA accurately and provide new ideas for the development of virtual QA and process optimization.

## Materials and Methods

### Data Collection

109 IMRT plans (including 568 fields) from December 2019 to May 2020 were selected. All plans were generated in the Eclipse version11 (Varian Medical Systems, Palo Alto, CA). Dose distributions were calculated using the Acuros External Beam (AXB, ver.11.0.31, Varian Medical Systems, Palo Alto, CA) with a dose calculation grid of 2.5 mm. Each plan was delivered using a linear accelerator equipped with a Varian Millennium 120 MLC. Patient-specific dose verification was performed prior to treatment using the actual angle by Portal Dosimetry. Daily dose calibration was performed during the period of data collection.

PD Mining, an in-house software developed by C #, was used to register, resample, and compare the calculated and measured dose distribution. This software was developed based on the interface of Varian ESAPI portal dosimetry and verified with the manual processing results. All patient dose data was searched through patient ID. The gamma analysis results and dose difference were obtained and exported to the local file in the form of text file automatically.

As 2%/2mm was the most sensitive criterion to detect clinically relevant errors (19, 20), it was used for establishing the classification model. If the GPR was higher than 85%, the field was considered to pass the QA, vice versa. The GPRs in the criteria of 3%/3mm, 3%/2mm, 2%/3mm were calculated at the same time. Absolute dose mode and 10% dose threshold were used for the above GPR analysis.

### Data Preprocessing

Pylinac library was used to extract the original image of planar dose distribution in the resolution of 1190 × 1190. Then the redundant information such as the frame and coordinate axis was cut off to get the image with the resolution 968 × 968. Flipping (horizontal random lip probability: 0.5, vertical random flip probability: 0.5) and random clipping were used to prevent overfitting. The images for the training set were randomly cropped from 968 × 968 to 960 × 960. The images in the test set were cut from the center, sizing from 968 × 968 to 960 × 960.

### UNet++ Architecture

Based on the traditional medical image processing network UNet, UNet++ enables the network to learn important features of different depths through a series of nested and dense jumping connections. By adopting deep supervision, UNet++ allows model complexity tuning to balance speed and performance optimization (21).

**Figure 1:**
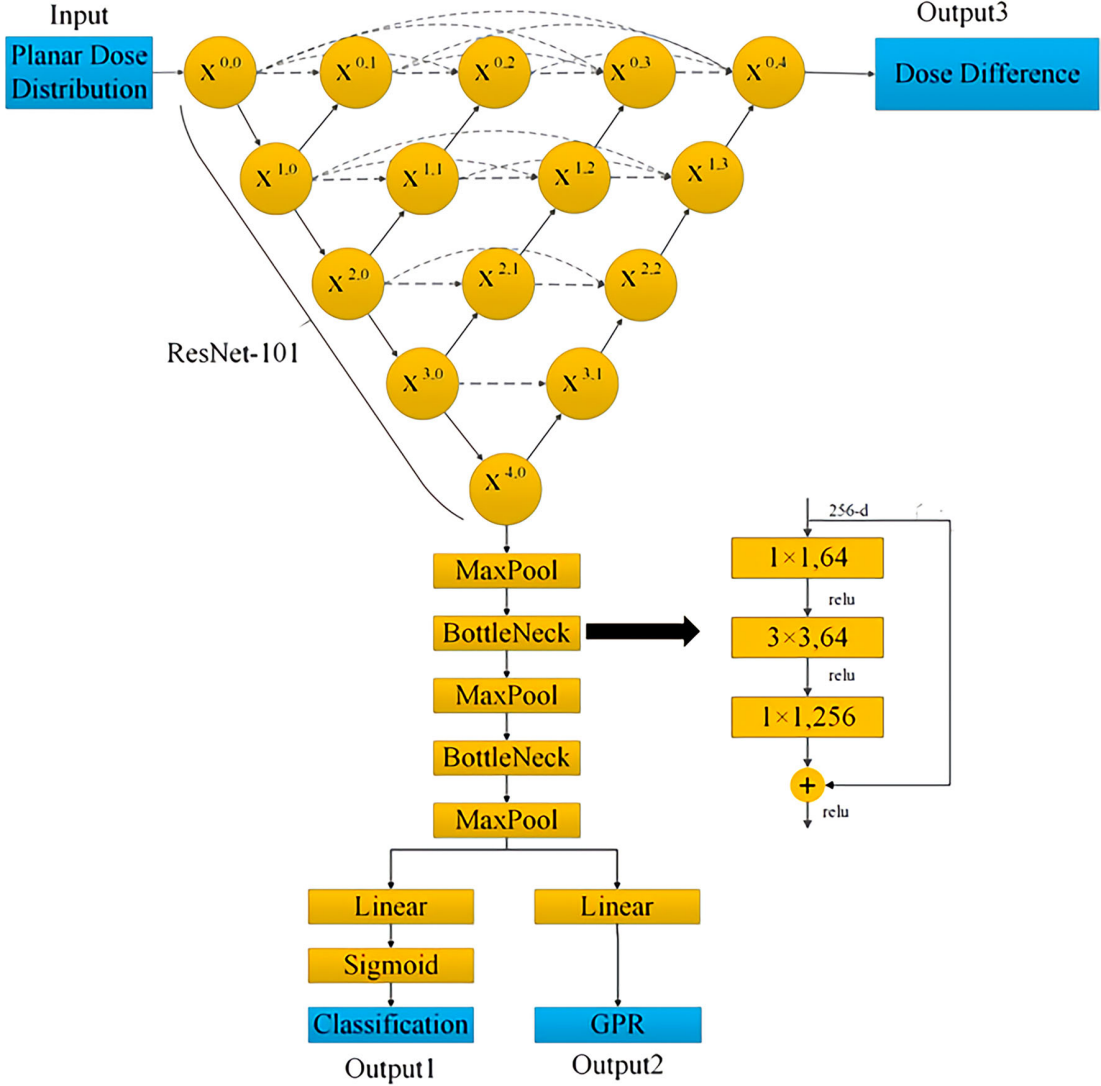
Architecture of the UNet++ used for prediction model.

**Figure 1** shows the architecture of the UNet++ used in this study. The downsample encoding channel of X^0,0^→X^1,0^→X^2,0^→X^3,0^→X^4,0^ adopted ResNet-101 architecture as the backbone network, and then the image size was restored by the corresponding upsample decoding nodes with skip connections. The planar dose distribution was input from X^0,0^, and the predicted dose difference image output of the same size was obtained from X^0,4^ after passing through the UNet++ network. At the same time, X^0,4^ was downsampled through three max-pooling layers and two bottleneck layers, and the linear layer was connected in series to predict the four gamma criteria GPR and the classification results.

### Model Training and Evaluation

To reduce dose difference prediction error caused by image scaling, the original resolution was adopted for the input and output. This processing would increase network memory and training time. Therefore, small batches rather than N-fold validation were selected. We randomly selected 95 fields (about 1/6) from the collected 568 samples as the test set. Four of the remaining samples (378 fields) were used as the training set and one as the validation set (95 fields).

The mean square error (MSE) loss function was used to evaluate the regression error of dose difference and GPR, and the binary cross-entropy was used to evaluate the classification error. The total loss was obtained by the weighted (1/3, 1/3, 1/3) sum of the three errors. The commonly used Adam optimizer (22) was adopted to learn the back-propagation error. The initial learning rate was set to 0.001, and it decreased exponentially as training going on, with the dropping rate setting to 0.9. A Mini batch method was used to train the model, the batch size was set to 2, and the epoch was set to 120. The prediction model was built by the open-source pytorch library. The entire training cost about 32 hours on the NVIDIA GTX-3080 GPU.

## Results

### Learning Curve for the Prediction Model

**Figure 2:**
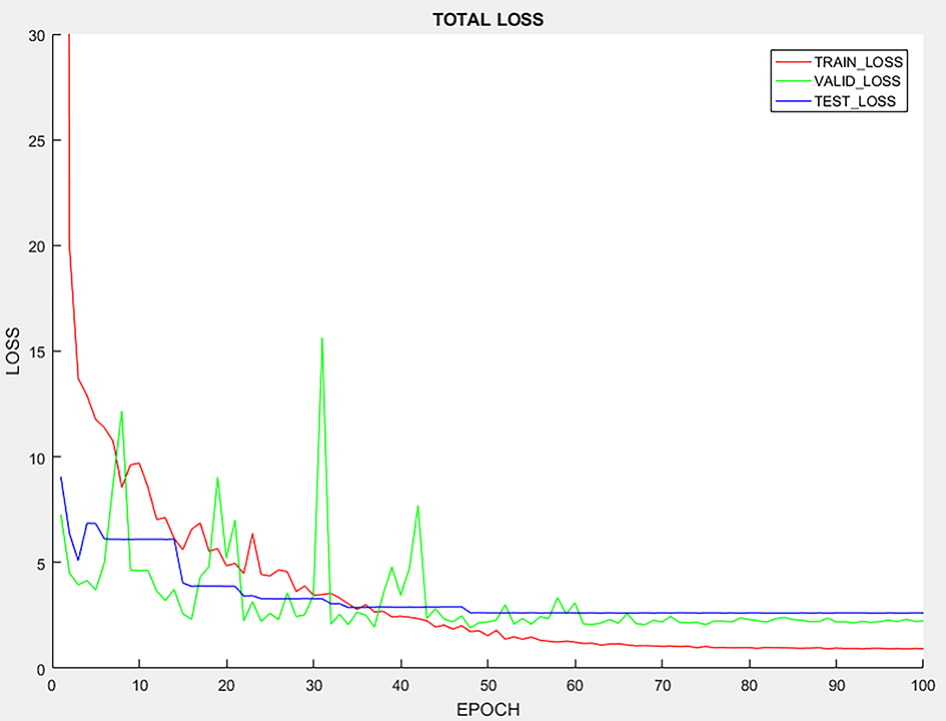
Learning curve of the prediction model.

It is expected that more epochs would give rise to higher prediction accuracy. Thus, there typically exists a minimum number of epochs beyond which the increase in prediction accuracy would saturate. **Figure 2** shows how the number of epochs will affect the accuracy for the prediction model. With more epochs, the loss on the training data, validation data and testing data decrease. With 60 epochs, the testing, validation and training loss converge at a stable level, indicating that increasing the epochs of training sets may not yield further improvement in the accuracy of the prediction model.

### Performance of Classification Model

The proportion of GPR less than 85% was 7.37% (7/95) and 8.42% (8/95) in the validation set and test set, respectively. As shown in **Table 1**, the sensitivity of the validation set is 57.14%, the specificity is 100%, and the accuracy is 96.84%. For the test set, the sensitivity is 62.50%, the specificity is 98.85%, and the accuracy of the classification model is 95.79%. From the analysis for the failed fields in measurement, while the prediction results are pass, the GPRs for these fields are near 85%, and the predicted GPRs are higher than 85%, so the classification results are pass fields.

**Table 1:** Results of classification model.

	Predicted-Fail	Predicted-Pass	
Validation set (95)			
Measured Fail	4	3	57.14%
Measured Pass	0	88	100.00%
Test set (95)			
Measured Fail	5	3	62.50%
Measured Pass	1	86	98.85%

The results are classified into four categories: failed measurement results and failed prediction results, TP; passed measurement results and passed prediction results (TN); passed measurement results and failed prediction results (FP); failed measurement results and passed prediction result passed (FN).

### GPR Prediction for Different Gamma Criteria

As shown in **Table 2**, the MAE and RMSE of the validation set and test set increase with stricter gamma criteria. In the test set, the smallest MAE and RMSE are 0.82 and 1.38 under 3%/3mm. The 2%/2mm gamma criteria has the largest MAE and RMSE in the test set, which are 2.52 and 3.72, respectively.

**Table 2:** MAE and RMSE for different gamma criteria.

	MAE		RMSE		
	Validation set	Test set	Validation set	Test set
3%/3mm	0.79	0.82	1.28	1.38
3%/2mm	0.93	0.88	1.50	1.57
2%/3mm	2.01	2.11	2.31	3.33
2%/2mm	2.17	2.52	3.00	3.72

**Figure 3:**
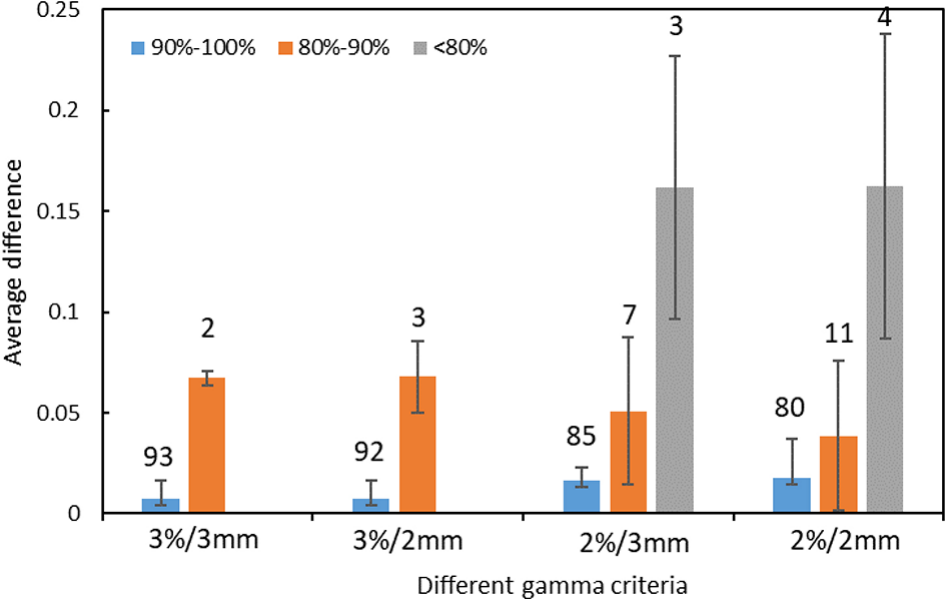
The distribution of prediction errors in test set among different groups under different gamma criteria. (Error bar: Mean ± standard deviation. The number on the vertical axis represents the number of fields for different groups).

The distribution of errors between the predicted GPR and the measured GPR under different gamma criteria are shown in **Figure 3**. The prediction errors among different gamma groups (90%-100%, 80%-90%, and < 80%) are compared. The accuracy of the prediction model is affected by the measured value itself. The higher the measured GPR, the smaller prediction errors between the measured and predicted GPR are observed.

### Dose Difference Prediction

**Figure 4:**
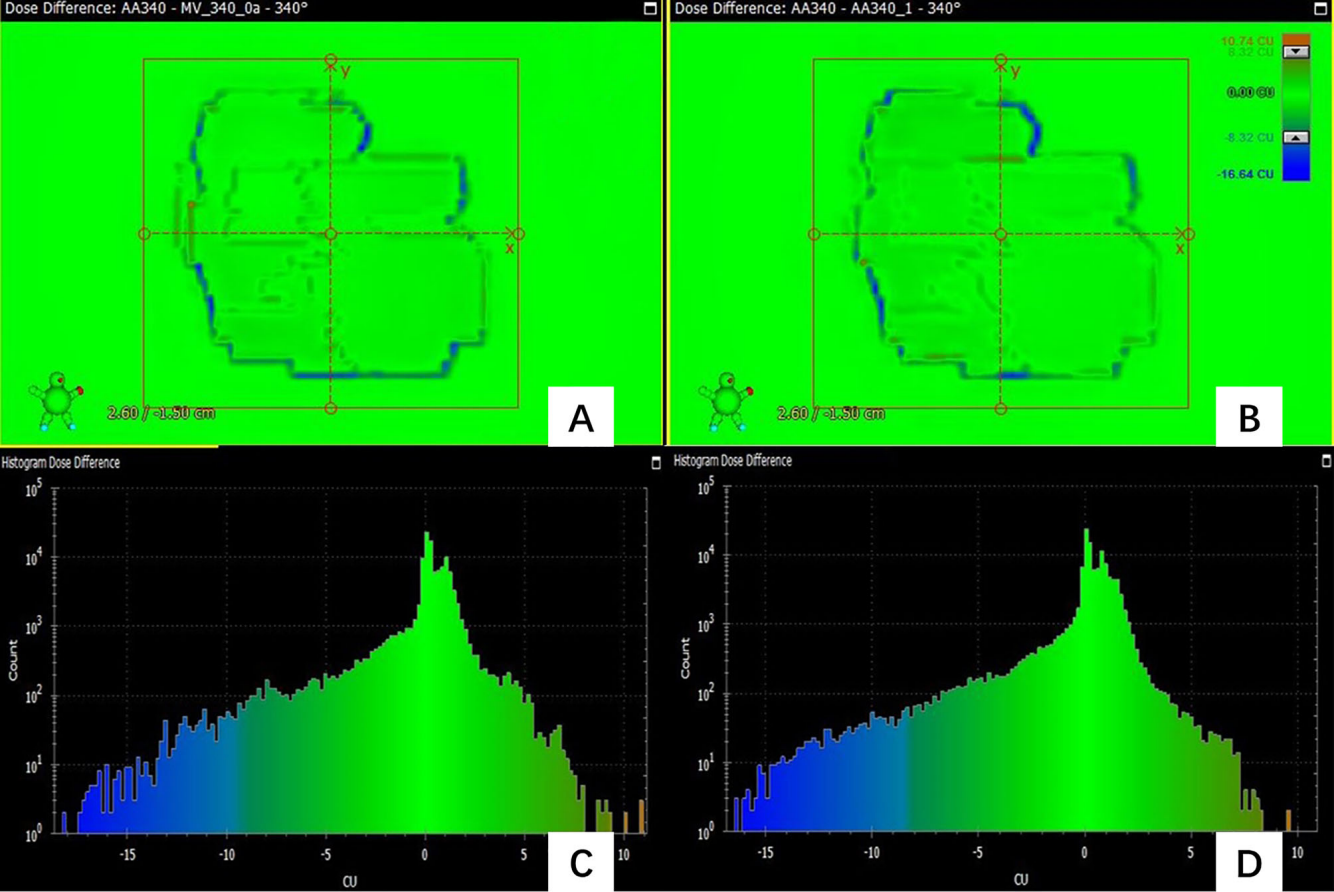
**(A)** the dose difference between the measured planar dose and TPS calculated **(B)** the dose difference between the predicted and TPS calculated planar dose. **(C)** the histogram of the dose difference between measured and TPS calculated planar dose for one pass field **(D)** the histogram of the predicted planar dose relative to TPS calculated dose.

**Figure 5:**
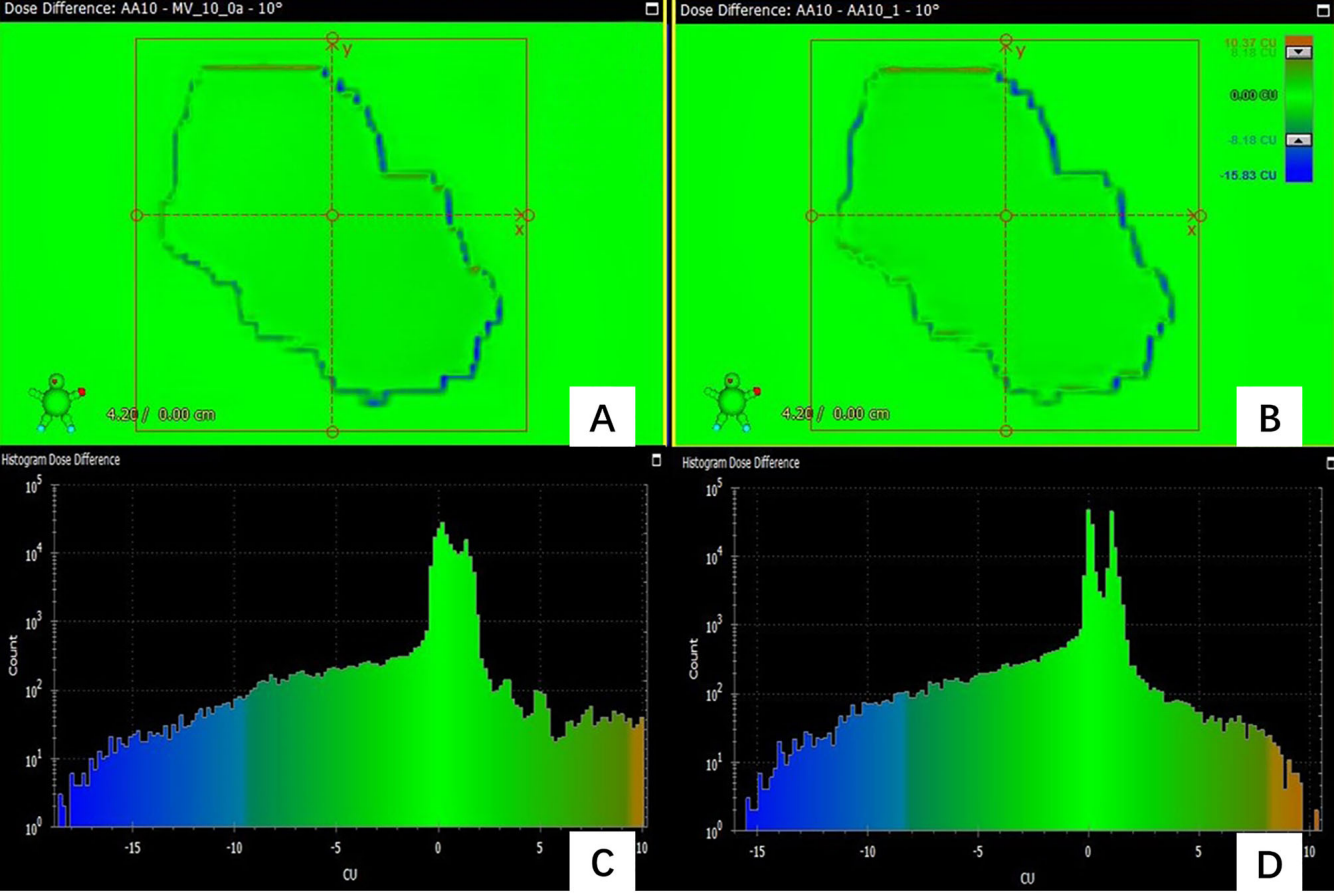
**(A)** the dose difference between the measured planar dose and TPS calculated **(B)** the dose difference between the predicted and TPS calculated planar dose. **(C)** the histogram of the dose difference between measured and TPS calculated planar dose for one failed field **(D)** the histogram of the predicted planar dose relative to TPS calculated dose.

In the prediction of dose difference, the dose difference and the histogram of distribution relative to TPS for the pass field and fail field are shown in **Figures 4**, **5**, respectively. The position and trend of predicted dose difference are consistent with the measured dose difference.

**Figure 4** shows the dose difference for a passing field of a patient. **Figure 4A** shows the dose difference between the measured planar dose and TPS calculated, and **Figure 4B** shows the dose difference between the predicted and TPS calculated planar dose. Even for a passing field, there are still some pixels with large dose difference, and their positions can be obtained. **Figure 4C** is the histogram of the dose difference between measured and TPS calculated planar dose for one failed field, and **Figure 4D** is the histogram of the predicted planar dose relative to TPS calculated dose. The histogram of predicted dose difference is consistent with the measured dose difference, the maximum and the minimum dose difference are almost the same.

As **Figure 5** shows, the predicted dose difference and measured dose difference for one of the failed fields and the histogram of dose difference. Large dose difference mainly located in the edge, and the measured dose is lower than the TPS calculated dose. The trend and position of the predicted dose difference is consistent with the measured dose difference, indicating that the virtual QA results could be used as guidance for the analysis of dose difference and plan redesign. By comparing and analyzing the dose difference for pass field and failed field, we found that the number of pixels having the large dose difference in the failed field was larger than in the pass field.

## Discussions

IMRT is a complex technology in radiotherapy, so special QA is required to ensure the accuracy of dose delivery. In this study, the UNet++ was used to classify QA results, GPR prediction of different gamma criteria and accurate dose difference prediction based on planar dose distribution. The accuracy of the classification model was 95.79%; there were small RMSE (1.38-3.72) and MAE (0.82-2.52) between the measured and the predicted GPR in the test set, and the trend and position of predicted dose difference were consistent with the measured dose difference. The results showed that the prospect of realizing virtual patient-specific QA with UNet++ and provides a new idea for the optimization of the individual QA process.

QA is required for every patient before treatment to ensure the accuracy of dose delivery (2–6). Dose verification depends on the equipment highly. The resolution and energy response of the detector will affect the result of dose verification. For the fields that fail the threshold, it is necessary to adjust the radiotherapy plan repeatedly, which will bring treatment delay. The process of dose verification is labor-consuming and time-consuming, so it brings more workload to the busy center. Automatic dose verification pre-measurement can mark the plans that fail the verification in advance and predict the dose difference, which is expected to be an effective method to solve the above problems.

As suggested in the TG 218 (6), the ability of the prediction model to accurately classify plans into “pass” or “fail” based on gamma criteria used is one of the most important indicators to evaluate the clinical feasibility of the model. The prediction of GPRs under different gamma criteria could provide more comprehensive information for physicists to judge whether the plan is acceptable clinically. The dose difference prediction model predicts the trend and location of dose difference, which provides direction for physicists to modify the plan. The paradigm shift of pre-measurement QA will improve the efficiency of dose verification greatly.

Since 2%/2mm is the most sensitive to clinically relevant errors (19, 20), it is selected as the basis of classification model in this study. The accuracy of failed fields was lower compared to pass fields, as the measured GPR were near 85% for some failed fields, and the predicted GPR were higher than 85%, so the predicted results of these fields were passed. Therefore, the selection of appropriate threshold plays an important role in the accuracy of the classification model and the proportion of failed fields needed to improve the accuracy of classification. The classification model gives the physicist a more intuitive result whether the field pass the QA pre-measurement.

The prediction model in this study can give the classification results according to the standard of the classification model, and also give the GPRs of different gamma criteria (3%/3mm, 3%/2mm, 2%/3mm, 2%/2mm). As an example, for one of the fail fields under the classification result, the GPR for 3%/3mm was 98.51% (Error: 0.01%), and GPR was 97.42% (Error: 0.22%) for 3%/2mm. After discussion by physicists and the doctors, the plan can be delivered. Therefore, the prediction of the GPRs of different gamma criteria could make a comprehensive judgment for the clinical enforceability of the plan.

In the prediction of GPR under different gamma criteria, the MAE and RMSE for the measured and predicted GPR of the model increase with the stricter gamma criteria, which is caused by the increase of the uncertainty of the prediction with the stricter gamma criteria. The accuracy of the model is affected by the measured GPR itself. The higher measured GPR is, the higher accuracy. This can be explained that the data with high measured GPR accounts for a large part of our model, so the accuracy of higher GPR prediction is high.

Previous studies only predicted the results of QA using machine learning or deep learning (9–18, 23, 24), but it is impossible to predict the trend and position of dose difference. The trend and position of dose difference plays a very important role in the analysis of the error source. However, there are no relevant reports about dose difference at present. In this study, prediction of dose difference based on the planar dose distribution is fulfilled. The result of this study that the predicted dose difference is consistent with the position and trend of the measured dose difference, can provide guidance for reason analysis.

It is noting that the pre-treatment prediction model established in this study is an auxiliary tool that needs a reparatory guarantee of the accuracy of the energy calibration and delivery process of the accelerator (2, 4). The purpose of this prediction model is not intended to replace the traditional QA, but to help physicists reduce the measurement burden of patient-specific QA when verifying the dose distribution and optimize the process of QA combined with other methods (25–28). There are still some limitations in this study: 1) As most of the clinical fields are pass fields, which leads to the unbalance of data distribution. Adequate amounts of low GPR plans for model training are needed to improve the accuracy of the model in future. 2) The model is limited to our data that all the data come from the same accelerator, the same energy, the same verification equipment. To expand the universality of the model, the research on models that include a variety of energy, different types of accelerators and dose verification equipment will be done in the future.

## Conclusion

In this study, we developed a UNet++ based prediction model for patient-specific QA. The prediction model could classify whether the field passes or fail QA, predict GPR of different gamma criteria, predict the trend and location of dose difference and mark the position. The virtual QA tool developed in this study provides a new idea for the optimization of patient-specific QA process, and promote the development of automated patient-specific QA.

## Data Availability Statement

The original contributions presented in the study are included in the article/supplementary material. Further inquiries can be directed to the corresponding author.

## Ethics Statement

Written informed consent was obtained from the individual(s) for the publication of any potentially identifiable images or data included in this article.

## Author Contributions

YH and YP: study concept and design and drafting of the manuscript. YH, KM, and RC: acquisition of data. YH and KM: analysis and interpretation of data. XM, SF, and JW: develop training model. HC, HW, HG, YS, YD, and AF: critical revision of the manuscript for important intellectual content. YH and SF: statistical analysis. WZ and ZX: study supervision. All authors contributed to the article and approved the submitted version.

## Funding

This study was funded by the Nurture projects for basic research of Shanghai Chest Hospital.

## Conflict of Interest

KM was employed by Varian Medical Systems. JW was employed by Ping An Healthcare Technology Co Ltd.

The remaining authors declare that the research was conducted in the absence of any commercial or financial relationships that could be construed as a potential conflict of interest.
